# A pilot study adapting and validating the Harvard Trauma Questionnaire (HTQ) and PTSD checklist-5 (PCL-5) with Indian women from slums reporting gender-based violence

**DOI:** 10.1186/s12905-022-01595-3

**Published:** 2022-01-28

**Authors:** Anushka R. Patel, Elana Newman, Julia Richardson

**Affiliations:** 1 Department of Psychology, The University of Tulsa, 800 South Tucker Drive, Tulsa, OK 74135 USA; 2 Department of Psychiatry and Behavioral Sciences, Trauma Recovery Center, University of California San Francisco, 2727 Mariposa St, San Francisco, CA 94110 USA

**Keywords:** Gender-based violence, Harvard Trauma Questionnaire, India, PCL-5, Idioms of distress, PTSD

## Abstract

**Background:**

Despite high rates of gender-based violence (GBV) in India, culturally sensitive measures that examine universal and culturally relevant trauma reactions are lacking. Although the Harvard Trauma Questionnaire (HTQ) has been used in India, no study has adapted the measure in full for use with this population. Similarly, the  PTSD checklist-5 (PCL-5) has not yet been validated in India. This study describes the adaptation, validation, and results from the adapted HTQ, and embedded PCL-5, for Indian women from slums reporting GBV.

**Method:**

This study used the adaptation framework proposed by the HTQ measure developers. The adapted HTQ contained a (1) trauma screen relevant for stressors faced by Indian women from slums, (2) description of the index trauma, (3) description of any ongoing stressors, (4) universal trauma reactions (i.e., PTSD measured by the PCL-5), and culturally relevant trauma reactions (i.e., idioms of distress measured by a scale developed for the study). This measure was piloted on 111 women from Indian slums in face-to-face interviews. Trauma characteristics, types of ongoing stressors, and psychometric properties of the PCL-5 and idioms of distress scale were explored. These scales were validated against measures of depression (PHQ-9), anxiety (GAD-7), and somatic complaints (PHQ-15).

**Results:**

The majority of participants (77%) reported physical beatings, 18% reported unwanted sexual touch, and 28.8% reported infidelity as the primary emotional abuse. Further, 96.7% of GBV was perpetrated by partner or family member and over half reported ongoing stressors (e.g., poverty-related strain). The PCL-5 embedded in the HTQ yielded good internal consistency (Cronbach’s alpha = .88) as did the idioms of distress scale with deletion of one item (Cronbach’s alpha = .80). Both scales were externally valid, yielding large correlations with depression, anxiety, and somatic complaints (*r*s between .54 and .80, *p*s < .05).

**Discussion:**

This is the first study to develop a comprehensive measure of trauma exposure with universal and culturally relevant trauma reactions in India. This study also enhances HTQ usage in India by delineating all the steps in the adaptation process. Results can inform the development of trauma-focused interventions for Indian women from slums.

**Supplementary Information:**

The online version contains supplementary material available at 10.1186/s12905-022-01595-3.

## Background

The gang rape of a New Delhi medical student in 2012 garnered global attention and catapulted India into national protests and discourse about gender-based violence (GBV) [1]. India has historically high rates of GBV [2–5] that disproportionately affect socioeconomically marginalized women with less education [6–8]. The risk of developing posttraumatic stress disorder (PTSD) following GBV is well established across cultures [9–11]. However, culturally relevant trauma reactions, such as idioms of distress, are comparatively under-studied following trauma exposure. PTSD and idioms of distress are especially relevant to study in low-and-middle-income countries (LMICs) as most psychological research originates from high-income countries (HICs) [12, 13]. Research from HICs may not generalize to LMICs as HICs comprise westernized, educated, industrialized, rich, and democratic peoples [12]. The current study thus investigates universal and culturally relevant trauma reactions (i.e., PTSD and idioms of distress) following GBV among Indian women from slums. Specifically, this study describes the adaptation and validation of the Harvard Trauma Questionnaire (HTQ) containing the PTSD checklist-5 (PCL-5) [14].

### Gender-based violence in India: conceptualization, prevalence, and gaps in scholarship

Gender-based violence in India is defined by physical, sexual, emotional, and economic abuses that typically occur within the family setting. National lifetime rates of GBV range from 8% for sexual violence [2] to 41% for multiple types of violence [5]. Significant regional variation exists, with rates up to 69% for lifetime GBV in certain Indian states [15]. Although GBV prevails across all demographic groups, women who are less educated and socioeconomically marginalized are at significantly greater risk of GBV compared to their educated and wealthier peers [5, 16–18].

Variation in the prevalence of GBV is partly attributable to how GBV is defined and assessed. Domestic violence (DV) is the most common type of GBV studied in India [19]. The WHO defines DV as “acts of physical, sexual, psychological abuse, and control by an intimate partner [or other member of the household] during a woman’s lifetime.” A recent study interviewing key informants in India described the principal characteristics of DV: controlling a woman’s reproductive decision-making, mobility, and socialization; tacit acceptance of sexual abuse; normalization of physical abuse through the presence of witnesses; psychological abuse over infertility, dissatisfaction with dowry and bearing girl-children; and perpetrating violence by the husband and his family members [19]. As the WHO’s definition of DV was limited in India, there is a need for comprehensive and culturally specific measurement of how DV—and other types of GBV—manifest in India.

According to a systematic review of 137 studies on GBV from India, 89% of studies focused on DV, excluding other forms of GBV [5]. Only 11% of studies used culturally adapted assessments, while the remainder relied on assessments validated in HICs. Only 12% examined psychological impacts of GBV and no studies systematically examined idioms of distress. Altogether, findings suggest four areas of improvement: (1) examine all types of GBV comprehensively, (2) assess culturally relevant manifestations of GBV in India (e.g., violence by in-laws), (3) investigate universal trauma reactions (i.e., PTSD) and (4) culturally relevant trauma reactions (i.e., idioms of distress).

### Trauma reactions: scholarship on PTSD and idioms of distress in India

While studies evaluate depression, anxiety, and suicide risk among Indian women [17, 20–22], few have explicitly examined trauma reactions following GBV among adult women [23]. PTSD is a common outcome of GBV [24]. Two studies in India found evidence for PTSD using the DSM-IV-TR. One study sampled treatment-seeking South Indian women reporting intimate partner violence [25] and another documented PTSD among sex workers reporting workplace violence [26], but neither study examined culturally relevant trauma reactions such as idioms of distress.

Research demonstrates that PTSD symptoms are cross-culturally universal, although the endorsement rate of symptoms varies considerably by cultural setting [25]. As such, idioms of distress offer a lens into cultural manifestations of distress. Idioms of distress are locally shaped and culturally recognizable forms of suffering [26–29]. Idioms of distress are not included in psychiatric diagnostic criteria precisely due to their local—rather than universal—relevance. Comprehensive trauma assessment, therefore, includes universal and culturally relevant trauma reactions.

Studies from India highlight three idioms of distress that are not incorporated by the diagnostic criteria of trauma and stressors-related disorders. First, Indian women complain of ‘*safed pani’* (white water), which refers to complaints of abnormal vaginal discharge and is associated with psychosocial factors such as marital strife and GBV [29–32]. Second, Indian women report ‘tension’—an idiom documented in many South Asian communities—which refers to negative mood states such as depression and anxiety, varied somatic complaints, and social difficulties [20, 21, 34, 35]. Finally, many studies from India reveal that women report diffuse somatic complaints, such as aches and pains, weakness, and dizziness associated with depression and anxiety following GBV [34–37].

Altogether, scholarship from India documents high rates of GBV and it is associated with depression, anxiety, and somatic complaints. Yet, few studies explicitly examine how GBV is associated with trauma reactions such as PTSD. To our knowledge, no published studies examine how GBV is associated with culturally relevant trauma reactions, such as idioms of distress. Scholars have called for culturally sensitive assessments to characterize trauma reactions more accurately in India [23, 38]. Cross-cultural assessments, such as the HTQ, are primed to fill this gap by querying trauma reactions that are universal (i.e., PTSD) and culturally relevant (i.e., idioms of distress).

### Cross-cultural assessment of trauma reactions: the HTQ and its use in India

The HTQ is a PTSD assessment originally developed for refugee populations [39]. It has since been translated and validated in several languages for many regions [40–47]. The HTQ is uniquely designed for cross-cultural use because it assesses PTSD according to mainstream diagnostic criteria plus idioms of distress that are typically excluded from standardized diagnostic schemes. The HTQ has evolved over the years and later versions [48] have four parts. Part I queries exposure to potentially traumatic events (PTEs) and stressors, Part II elicits a description of an index trauma and any ongoing stressors, Part III assesses traumatic brain injury, and Part IV measures symptoms in two categories: the 16 PTSD symptoms corresponding to the DSM-IV-TR and locally relevant idioms of distress. The HTQ has an additional strength in that it documents ongoing stressors, which are common contextual factors in LMICs that require careful assessment for appropriate diagnostic and treatment planning [49, 50].

One study from India sampled adults in Kashmir Valley to validate Part IV of the HTQ against the psychiatrist-administered MINI using one broad screening question for trauma exposure [51]. They found high internal consistency reliability for the HTQ-IV (Cronbach’s alpha = 0.90) but they could not validate the measure externally against a gold-standard PTSD interview because only 3.4% of PTSD cases were diagnosed. Another study from India sampling youth also examined the HTQ-IV [52]. They also found high internal consistency reliability for the HTQ-IV (Cronbach’s alpha = 0.90). Altogether, both studies from India are limited by use of only a section of the HTQ rather than the whole measure, outdated symptoms corresponding to the DSM-IV-TR, no assessment of idioms of distress, no information on whether participants experienced ongoing stress, or whether trauma reactions were explicitly linked to the trauma [51, 52]. Further, the HTQ symptom domains need to be updated for the current classification in the *DSM-5*, which features 20—rather than 16—symptoms.

An alternative to the HTQ-IV is the PTSD checklist-5 (PCL-5). The PCL-5 is a self-report measure assessing 20 symptoms of PTSD according to the *DSM-5* [14]. The PCL-5 yields strong diagnostic convergence with clinician-administered interviews for PTSD [53] and is a well-established reliable and valid instrument [54–56]. Only a few studies have examined its psychometric properties in LMICs [57, 58] and none have adapted the PCL-5 into Hindi.

## Study aims

The current study addresses the specific gaps of prior HTQ studies in India by detailing the adaptation and validation of the full HTQ including the PCL-5 embedded into it. This study has three major aims: (1) Document the prevalence of multiple types of GBV; (2) Establish the reliability of two subscales of the HTQ-IV (i.e., idioms of distress and PCL-5); and (3) Establish the external validity of the two subscales of the HTQ-IV (i.e., idioms of distress and the embedded PCL-5 by examining their relationships with known correlates such as depression, anxiety, and somatic problems [25, 35, 59, 60].

##  Methods

### Recruitment procedure and setting

This study used community-based participatory research principles by partnering with a grassroots non-governmental organization called CORO for Literacy that provided educational, social, and legal programming for under-served slum communities in Mumbai. Participants were recruited through referral sampling, whereby organization members referred participants to the researcher, who is Indian, female, bilingual, and had three years of clinical interviewing experience. All meetings were conducted in private rooms to protect participants’ privacy. Participants were orally administered consent forms so they could make informed decisions about participation. They were also reminded of the confidential and voluntary nature of participation, limits to confidentiality, and the risks and benefits of study participation. Participants between ages 18–65 who provided verbal and written informed consent in Hindi and endorsed at least one item on the trauma screen (HTQ-I) were included in the study. All study procedures were approved for ethical research conduct by an institutional review board at an American university and by CORO for Literacy. Data were collected in two waves: June to August 2015 and August to October 2018.

### Study procedure and study sample

Data were collected through verbal administration due to low literacy rates in Indian slums [61]. Participants were administered a brief demographic form, followed by a PTEs and stressors (HTQ-I) screen. In total, 111 participants completed the HTQ-I and were included for the first aim; of the 111 participants, the 99 who were able to link their PTSD symptoms and idioms of distress to an index trauma were included for analysis of the second and third aims. Administration of HTQ-I was followed by a brief description of their index traumatic event (HTQ-II), any ongoing stressors (HTQ-III), and symptom reports on the PCL-5 (HTQ-IVa) and idioms of distress scale (HTQ-IVb). Lastly, participants were administered validated screeners for depression, anxiety, and somatic problems using the Patient Health Questionnaire (PHQ) series.

### Adapted Harvard Trauma Questionnaire (HTQ)

**Fig. 1:**
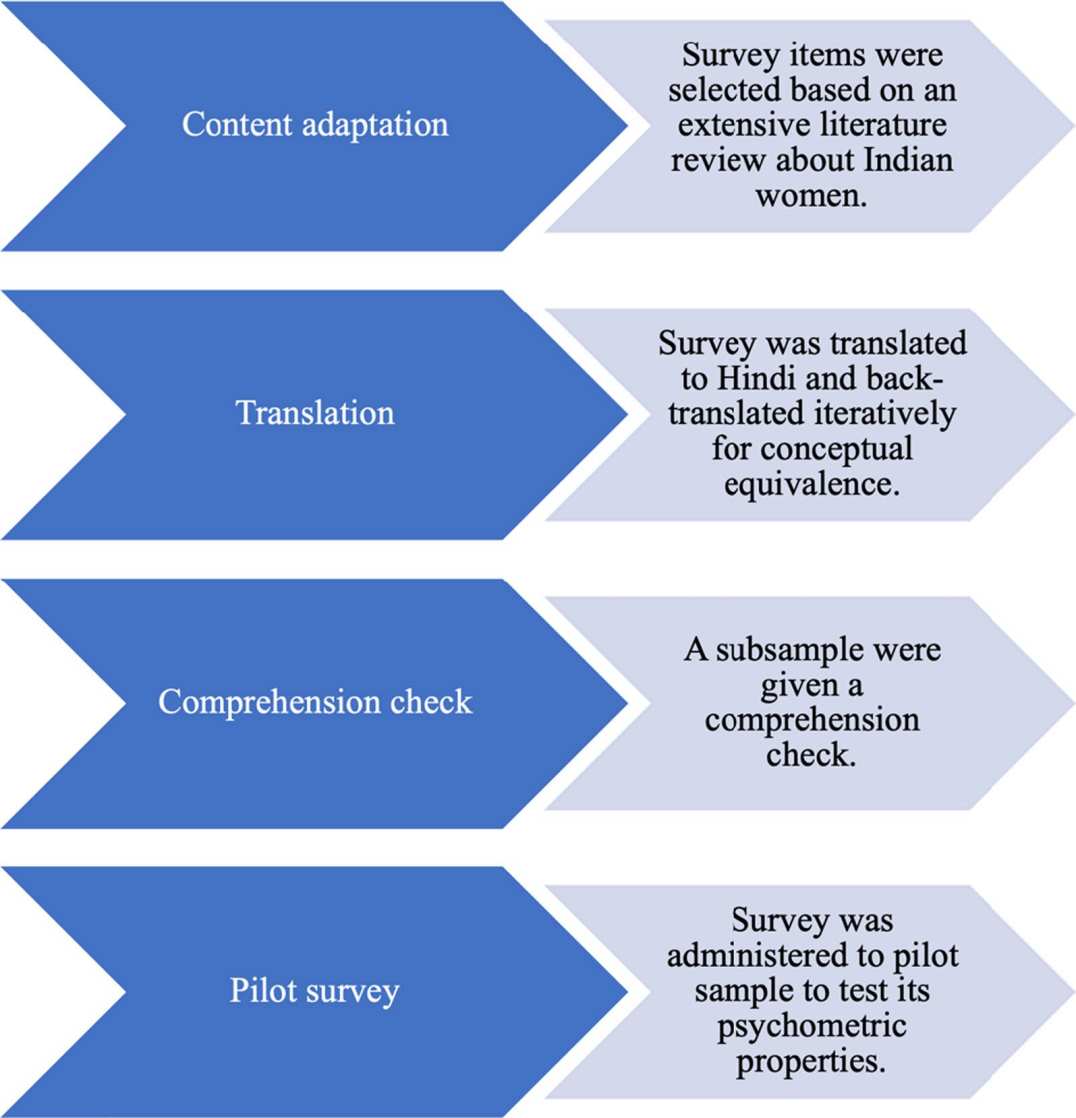
Overview of adaptation procedures for the Indian HTQ

**Fig. 2:**
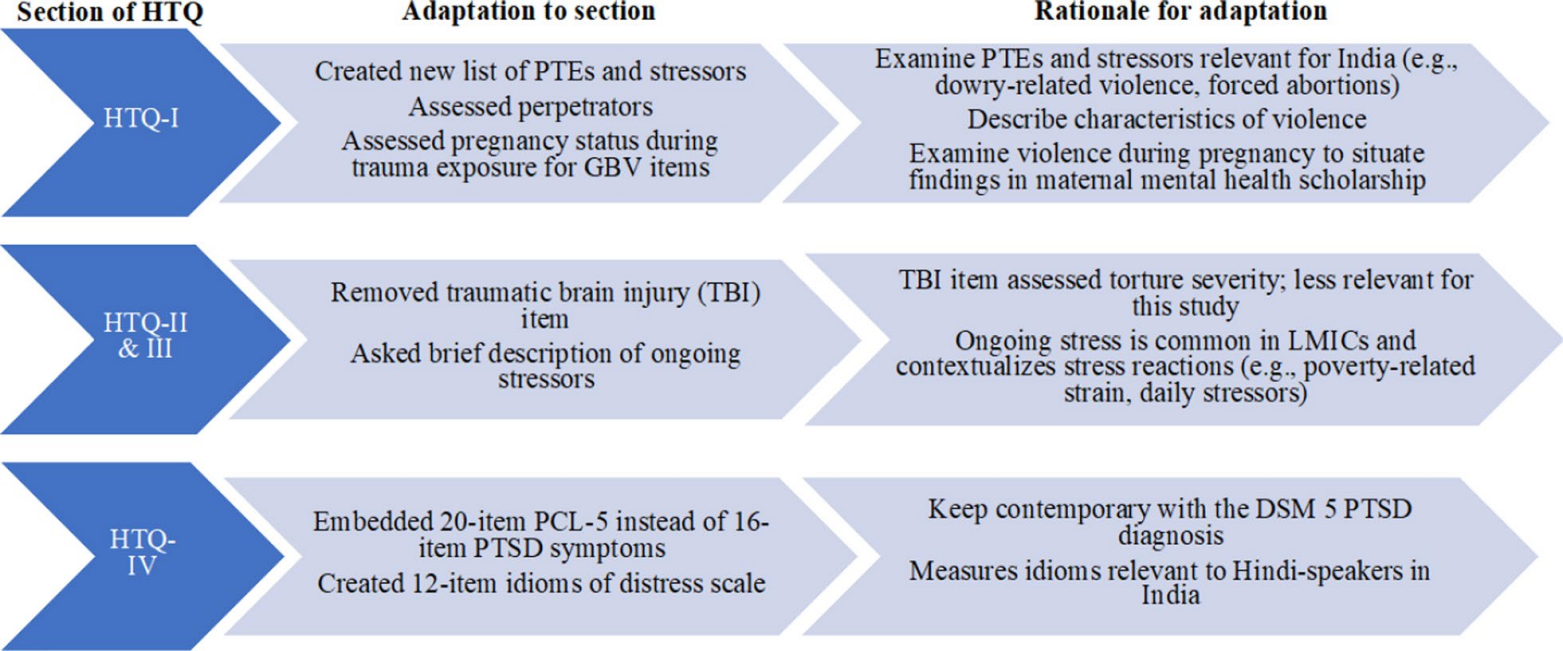
Adaptations, and rationale, for the Indian HTQ

The HTQ was adapted following the established procedures of the measure developers in a recent study of Iraqi refugees [62]. These methods were consistent with recommendations in cultural epidemiology for assessment tool adaptation [62, 63]. The content adaptation of the HTQ is described below by section, followed by adaptation procedures for translation. Figure 1 provides an overview of the adaptation procedures. For a global view of all adaptations by section, see Fig. 2 and Additional File 1.

### Adaptation of HTQ-I: PTEs, stressors, and perpetrators

The HTQ was adapted to assess PTEs and stressors relevant in India by explicitly querying for PTEs that included GBV and non-GBV stressors. The PTEs and stressors were created from an extensive literature review about Indian women [5]. The PTEs and stressors consistent with physical and sexual GBV include domestic violence perpetrated by partners and family members [5], sex trafficking [64], and dowry-related violence [65]. Information on the perpetrators of GBV events was also collected by asking participants to select one or more perpetrators for each event from partner, family/in-laws, friend/acquaintance, and/or other. Similarly, as Indian women experience violence during pregnancy [66], pregnancy status was obtained for GBV events.

### Adaptation of HTQ-II and HTQ-III: index trauma and ongoing stressors

Participants were asked to briefly describe their index trauma. All symptom reports in HTQ-IV were linked to this index trauma to keep consistent with the intended administration of the PCL-5. Participants were also asked: “Under your current living situation, what is the worst event that has happened to you, if different from above. Please specify where and when these events occurred.” Responses to this question were recorded as descriptive information and also coded dichotomously as ‘0’ (when participants endorsed no ongoing stress) and ‘1’ (when participants endorsed ongoing stress).

### Adaptation of HTQ-IV: Symptom domains of HTQ

The HTQ was adapted to assess three types of trauma-related symptoms after participants endorsed a PTE or stressor. These included 20 PTSD symptoms in the *DSM-5* derived from the PCL-5 [14] and 12 idioms of distress specific to India and created for this study.

#### HTQ-IVa: Hindi PCL-5 within the HTQ

A 20-item checklist, the PCL-5 assesses PTSD symptoms from the *DSM-5* [14]. Each symptom is rated for severity on a 5-point Likert scale ranging from 0 to 4, so total scores could range from 0 to 80. All participants were asked to anchor their symptoms to their most stressful event in the past 30 days. The Hindi PCL-5 was scored continuously. Any PCL-5 symptoms endorsed at or above moderate (‘2’) levels (i.e., rating of ‘2’, ‘3’, or ‘4’) were considered clinically elevated symptoms [14].

#### HTQ-IVb: idioms of distress within the HTQ

A 12-item checklist, the idioms of distress scale contains idioms of distress gleaned from literature review and during discussions with two Indian key informants who were bilingual and familiar with trauma-related reactions. Examples of idioms included somatic complaints, such as orthostatic dizziness and pain in the nerves; culturally-laden cognitions, such as believing that the trauma was written in one’s destiny; and affective reactions, such as tension. Each symptom is rated for severity on a 4-point Likert scale ranging from 1 to 4, so total scores could range from 12 to 48. As this study did not validate the idioms of distress scale against a clinician-administered measure, the level at which symptoms were clinically elevated could not be determined.

#### Adaptation procedure: translation process and comprehension check

Consistent with other cross-cultural assessment tool adaptation, steps to translate and back-translate the instrument to ensure technical, semantic, and conceptual equivalence were undertaken [63]. The assessment tool was first translated into Hindi, then back-translated into English by two independent bilingual individuals. For the PCL-5 measure embedded into HTQ-IV, two English-speaking PTSD experts rated the original and back-translated versions for conceptual equivalence on a 5-point Likert scale (ranging from ‘0’ denoting it was not at all the same meaning to ‘5’ denoting it was exactly the same meaning). Items rated at or below 3 were then semantically adjusted iteratively so the final back-translated version reflected the original meaning before being re-instated into the final Hindi HTQ.

As the wording of PTSD symptoms may not have been comprehensible to participants who may not be familiar with PTSD in this cultural context, the Hindi PCL-5 was also subjected to a comprehension check. A subsample (12%) were administered the comprehension check. For items the subsample endorsed (rated ‘1’, ‘2’, ‘3’, ‘4’), they were asked to provide examples of the symptoms. For the items the subsample did not endorse (rated ‘0’), participants were asked to explain the meaning of the prompt to ensure conceptual clarity. Answers were recoded verbatim and later coded by both authors against the prompts on the CAPS-5, a gold-standard clinician-administered PTSD assessment. Results of the coding process and disagreements were resolved through discussion and consensus.

### Patient health questionnaire-9 (PHQ-9)

A 9-item screening instrument, the PHQ-9 [67] assesses the presence and severity of current depression and has been validated for use in India [68]. This questionnaire queries all depression symptoms with response options ranging from 0 (‘not at all’) to 3 (‘nearly every day’) in the past two weeks, with total scores ranging from 0 to 27. The PHQ-9 demonstrated adequate internal consistency reliability in this study (Cronbach’s α = 0.78).

### Generalized anxiety disorder screen (GAD-7)

A 7-item screening instrument, the GAD-7 [69], assesses the presence and severity of anxiety (including generalized anxiety, panic, and social anxiety) and has been validated in India [68]. The GAD-7 has response options ranging from 0 (‘not at all’) to 3 (‘nearly every day’) in the past two weeks and total scores range from 0 to 21. The GAD-7 demonstrated good internal consistency reliability in this study (Cronbach’*s* α = 0.84).

### Patient health questionnaire-15 (PHQ-15)

A 15-item screening instrument, the PHQ-15 [70], assesses the presence and severity of somatic complaints such as headaches, menstrual cramps, indigestion, and pain during sex. Response options range from 0 (‘not bothered at all’) to 2 (‘bothered a lot’) over the past two weeks and total scores range from 0 to 30. It has been used in India in studies with women experiencing depression [71, 72]. The PHQ-15 demonstrated adequate internal consistency reliability in this study (Cronbach’*s* α = 0.79).

## Results

### Participant characteristics

Participants were 34.13 (*SD* = 9.64) years old on average. They completed an average of 6.97 (*SD* = 3.76) years of education and subsisted on an average monthly per capita income of 57.99 USD (*SD* = 61.06 USD). The majority of participants were married (78.57%), of Dalit ethnicity (42.86%), and originated from a city (69.39%).

### HTQ-I and II: potentially traumatic events (PTEs) and ongoing stressors

On average, participants endorsed 3.97 (*SD* = 2.36) GBV events, 1.12 (*SD* = 0.94) non-GBV events, and 5.09 (*SD* = 2.80) total traumatic events. Nearly all (96.91%) reported that GBV events were perpetrated by their partners, family members, or in-laws and 62.92% of participants selected a GBV event as their index trauma. The most common GBV events were being kicked or beaten, directly witnessing others being kicked or beaten, and directly witnessing others attacked with a weapon. The prevalence of each Criterion A event surveyed can be found in Table 1. Participants also endorsed culturally relevant GBV, such as dowry-related violence and sex trafficking, respectively. The most common non-GBV events were the sudden loss of a loved one, followed by a major accident, and surviving a natural or manmade disaster.

**Table 1:** GBV and other Criterion A event prevalence from the adapted Hindi HTQ (*N* = 111)

Trauma type	%	*n*
*Interpersonal physical violence*		
Been hit, beaten or kicked?	77.5	86
Witnessed someone being hit, beaten or kicked?	60.4	67
Witnessed someone being hit with an object/weapon/something thrown at them (e.g. boiling water or acid)?	30.6	34
Been threatened with violence?	29.7	33
Witnessed someone being threatened by violence?	25.2	28
Been hit with an object/weapon/had something thrown at you (e.g. boiling water or acid)?	24.3	27
Personally experienced a robbery or mugging?	18.9	21
Personally experienced police brutality when you did not commit a crime?	9.0	10
Been Involved in communal riots?	9.0	10
*Sexual violence*		
Had someone touch your private parts when you did not want them to?	18.0	20
Made to touch someone’s private parts when you did not want to?	11.7	13
Made to have a hysterectomy or abortion?	10.8	12
Made to have sex with someone against your will?	7.2	8
Contracted a sexually transmitted disease from unwanted sex?	5.4	6
Coerced into commercial sex work?	2.7	3
Had a physical or sexual assault that resulted in miscarriage or infant harm?	2.7	3
*Family-related and emotional violence*		
Learned about your husbamd having an affair?	28.8	32
Felt that someone else consistently made decisions for you?	26.1	29
Been ignored by a loved one for a long period of time?	20.7	23
Been consistently humiliated or made to feel small by someone?	20.7	23
Been called derogatory names for being a woman?	16.4	18
Been called derogatory names for bearing a girl child?	13.4	15
Experienced violence, coercion or bullying related to dowry?	9.1	10
Personally experienced a family member hurting your child in order to hurt you?	9.1	10
Were forced into an unwanted marriage?	5.4	6
*Bereavement or loss*		
Experienced loss of a loved one due to unexpected or violent death?	57.5	64
Experienced loss or destruction of home/land?	18.0	20
Experienced forced separation from a child?	13.6	15
Experienced forced separation from a family member?	9.9	11
*Physical and mass trauma*		
Survived a life-threatening medical illness?	21.6	24
Experienced starvation for an extended period of time?	18.0	20
Survived a serious accident (e.g. house fire or car accident)?	14.4	16
Survived a serious natural disaster (e.g. earthquake or flood)?	10.8	12
Other stressful experience?	0	0

Event endorsement was not mutually exclusive

### HTQ-III: ongoing stressors

More than half the sample (54.64%) reported ongoing stressors. Types of ongoing stressors included poverty-related stress (e.g., difficulty meeting household, childcare, education expenses), caregiving stress (e.g., getting children married, caring for sick family members), and emotional abuse (e.g., humiliation by in-laws, marital infidelity, desertion). Notably, 13 participants (i.e., 13.40%) reported that their ongoing stressor was a traumatic event that met the definition of Criterion A (e.g., domestic violence, marital rape).

### HTQ-Iva: psychometric properties of the Hindi PCL-5

The full-scale reliability of the total PCL-5 scale was good (Cronbach’s α = 0.88). The mean inter-item correlation for the full-scale PCL-5 was 0.27, indicating a satisfactory item agreement. The mean full-scale Hindi PCL-5 score was 29.88 (*SD* = 17.06). Table 2 delineates mean scores per item.

**Table 2:** Descriptive statistics, psychometric properties, and symptom frequency of the Hindi PCL-5 (N = 99)

Item	*M*	*S*D	Cronbach's Alpha if Item Deleted	Corrected Item-Total Correlation	Skew	% endorsement
Intrusive memories	2.26	1.50	0.88	0.51	− 0.90	62.00
Psychological reactivity	2.33	1.42	0.88	0.57	− 0.44	62.00
Persistent negative emotions	1.95	1.62	0.87	0.73	0.22	54.00
Physical reactivity	1.73	1.54	0.88	0.52	0.87	51.00
Hypervigilance	1.79	1.54	0.89	0.04	0.87	50.00
External avoidance	1.77	1.58	0.88	0.54	1.02	47.00
Internal avoidance	1.68	1.48	0.88	0.52	1.34	46.00
Irritability	1.62	1.67	0.88	0.53	1.49	46.00
Startle reflex	1.60	1.52	0.88	0.55	1.78	45.00
Difficulty with sleep	1.51	1.65	0.88	0.52	1.88	43.00
Distorted blame	1.37	1.59	0.88	0.51	2.44	39.00
Negative beliefs	1.75	1.71	0.87	0.59	1.07	37.10
Detachment	1.22	1.60	0.87	0.62	3.32	34.00
Inability to have positive emotions	1.34	1.64	0.88	0.54	2.95	33.00
Diminished interest	1.10	1.49	0.88	0.39	4.00*	30.00
Trauma-related nightmares	0.99	1.37	0.88	0.49	4.61*	27.00
Flashbacks	0.99	1.41	0.88	0.44	4.88*	25.00
Self-destructive behavior	0.72	1.35	0.88	0.54	6.61*	21.00
Difficulty concentrating	1.56	1.60	0.88	0.60	1.36	17.50
Trauma-related amnesia	0.62	1.22	0.89	0.03	8.17*	14.00

*M* mean, *SD* standard deviation

*p* < .05

*Indicates item is significantly positively skewed

Intrusive memories, psychological reactivity, and persistent negative emotions were most frequently endorsed at clinically elevated levels and yielded the highest mean item scores. Conversely, trauma-related amnesia, self-destructive behaviors, and flashbacks were the least frequently endorsed and yielded the lowest mean item scores. Notably, the items for diminished interest, trauma-related nightmares, flashbacks, self-destructive behaviors, and trauma-related amnesia were significantly positive skewed. The internal consistency of the Hindi PCL-5 could be improved by deleting trauma-related amnesia and hypervigilance (see Table 2).

Twelve participants’ comprehension of individual PCL-5 items was assessed. The subsample, who were interviewed about each PCL-5 item, appeared to understand the meaning of most items barring two items (see Table 3).

**Table 3:** Percentage of participants who comprehended the Hindi PCL-5 items (n = 12)

Item	% comprehension	*n*
Repeated, disturbing and unwanted memories of the stressful experience?	100	12
Repeated disturbing dreams of the stressful experience?	100	12
Feeling very upset when something reminded you of the stressful experience?	100	12
Having strong physical reactions when something reminded you of the stressful experience (for ex. Heart pounding, trouble breathing, sweating)?	100	12
Avoiding internal reminders of the stressful experience (ex. Thoughts, feelings or physical sensations)?	100	12
Avoiding external reminders of the stressful experience (ex. People, places, conversations, objects, activities or situations)?	100	12
Trouble remembering important parts of the stressful experience?	100	12
Having strong negative feelings such as fear, horror, anger, guilt or shame?	100	12
Loss of interest in activities that you used to enjoy?	100	12
Having trouble experiencing positive feelings (ex. Being unable to have loving feelings for those close to you, or feeling emotionally numb)?	100	12
Feeling distant or cut off from people?	100	12
Feeling irritable or angry or acting aggressively?	100	12
Being 'superalert' or watchful or on guard?	100	12
Feeling jumpy or easily startled?	100	12
Having difficulty concentrating?	100	12
Blaming yourself or someone else strongly for the stressful experience or what happened after it?	92	11
Trouble falling or staying asleep?	92	11
Taking too many risks or doing things that cause you harm?	83	10
Having strong negative beliefs about yourself, other people, or the world (ex. Having thoughts such as: I am bad, there is something seriously wrong with me, no one can be trusted, the world is completely dangerous)?	75	9
Suddenly feeling or acting as if the stressful experience were actually happening again (as if you were back there reliving it?)	58	7

Specifically, flashbacks were difficult to understand for those participants who had never experienced them. This item resulted in the lowest comprehension (58.33% comprehension rate). Participants who misunderstood flashbacks typically reported general difficulty with understanding it or described ruminative symptoms instead. Similarly, globalized negative beliefs about oneself, the world, and others also proved problematic to comprehend (75% comprehension rate). Participants typically misunderstood this item to mean wishing ill of others and were quick to deny this reaction.

### HTQ-IVb: psychometric properties of the idioms of distress scale

The full-scale reliability of the idioms of distress scale was acceptable (Cronbach’s α = 0.79). The mean inter-item correlation for this scale was 0.23, indicating a satisfactory item agreement. The mean idioms of distress scale score was 24.59 (*SD* = 7.11). Table 4 delineates mean scores per item. Tension, attributing the trauma to destiny, and heat in the head/headaches yielded the highest mean item scores. Conversely, *safed pani* (vaginal discharge), *nas mein dard* (pain in the nerves), and attributing the trauma to being a woman were the least frequently endorsed and yielded the lowest mean item scores. Most items appeared to be worth retaining as deletion would result in a decrease in the scale’s reliability. However, deleting the item, “Believing that you deserved what happened because you are a woman,” would increase the scale’s Cronbach’s α from 0.79 to 0.81 (see Table 4). Notably, six items on the idioms of distress scale were significantly positively skewed: Fear of others learning about this hurtful experience, feeling that you are a burden on your family, feeling that you brought shame on your family, *safed pani* (vaginal discharge), *nas mein dard* (pain in the nerves), believing that you deserved what happened because you are a woman. These items were endorsed less frequently and with less intensity in this sample. Barring one item—“believing that you deserved what happened because you are a woman,”—the idioms of distress scale retained good reliability by including the remaining skewed items.

**Table 4:** Descriptive statistics, psychometric properties, and symptom frequency of the idioms of distress scale *(N* = *99)*

Item	*M*	SD	Cronbach's Alpha if item deleted	Corrected item-total correlation	Skew
Tension	2.83	1.17	0.77	0.54	− 1.62
Believing that this was destined to happen and written in your stars	2.66	1.10	0.78	0.43	− 0.49
Sar mein garmi (heat in the head/headache)	2.59	1.06	0.78	0.46	− 0.02
Dil mein udasi (sadness in your heart)	2.51	1.18	0.76	0.63	0.06
Chakkar (dizziness)	2.14	1.05	0.77	0.55	2.32
Dizziness when standing up	2.07	1.09	0.77	0.52	2.55
Fear of others learning about this hurtful experience	1.90	1.18	0.77	0.50	3.50*
Feeling that you are a burden on your family	1.86	1.22	0.78	0.44	3.94*
Feeling that you brought shame on your family	1.70	1.16	0.78	0.42	5.34*
Safed pani (vaginal discharge)	1.42	0.73	0.79	0.35	6.52*
Nas mein dard (pain in the nerves)	1.55	1.03	0.79	0.27	6.74*
Believing that you deserved what happened because you are a woman	1.36	0.76	0.81	0.06	8.19*

*M* mean, *SD* standard deviation

*p* < .05

*Indicates item is significantly positively skewed

### Convergent validity of HTQ-IV: Hindi PCL-5 and idioms of distress scale

Posttraumatic stress disorder, idioms of distress, depression, somatic complaints, and anxiety yielded large positive correlations ranging from 0.54 (PTSD and somatic complaints, *p* < 0.001) to 0.80 (PTSD and idioms of distress, *p* < 0.001) (see Table 5).

**Table 5:** Correlation matrix of PTSD, idioms of distress, depression, somatic complaints, and anxiety

	PTSD	Idioms of distress	Depression	Somatic complaints	Anxiety
PTSD	–	–	–	–	–
Idioms of distress	.80**	–	–	–	–
Depression	.68**	.75**	–	–	–
Somatic complaints	.54**	.60**	.66**	–	–
Anxiety	.73**	.75**	.77**	.61**	–

**Significance level of *p* < .01. PTSD symptoms were measured with the Posttraumatic Stress Disorder Checklist-5 (PCL-5) (Weathers et al. [14]). Idioms of distress were measured by the subscale created for the study on the Harvard Trauma Questionnaire (HTQ). Depressive symptoms assessed by the Patient Health Questionnaire—9 (PHQ-9) (Kroenke et al. [67]). Somatic complaints were measured by the Patient Health Questionnaire—15 (PHQ-15) (Spitzer et al. [69]). Anxiety was measured by Generalized Anxiety Disorders-7 (GAD-7) (Spitzer et al. [69])

## Discussion

This study sought to adapt and validate the HTQ for Indian women from slums exposed to GBV. The trauma screen measured a wide range of GBV and non-GBV events, indicating high levels of trauma exposure across traumatic event categories in this sample. The Hindi PCL-5, embedded to measure universal trauma reactions of PTSD, appeared to be psychometrically useful and valid, although the hypervigilance and trauma-related amnesia items did not appear to measure PTSD. The Hindi PCL-5 demonstrated high convergence with measures of depression, anxiety, and somatic complaints. The idioms of distress scale, developed to assess culturally relevant reactions, also appeared to have promising psychometric properties. This scale yielded good reliability with the deletion of one item (i.e., believing that you deserved what happened because you are a woman) and also demonstrated convergent validity with known correlates of PTSD.

### Reliability, validity, and comprehension of the Hindi PCL-5

Results indicated that the Hindi PCL-5 demonstrated good reliability estimates, adding to a body of literature from HICs [73–75] and LMICs [41, 76–81]. The Hindi PCL-5 yielded a very similar full-scale reliability to the 17-item PCL adapted for India in an older study (i.e., Cronbach’s α = 0.89) [82]. Overall, the full-scale reliability of the Hindi PCL-5 indicates that it assessed multi-faceted aspects of PTSD while also reliably measuring overall PTSD symptoms.

Similarly, results support the validity of the Hindi PCL-5. Medium-to-large positive correlations between full-scale PTSD, depression, and anxiety indicate that the Hindi PCL-5 assessed a construct that was related to depression and anxiety in the expected direction and magnitude. These findings align with the validity literature from HICs [53, 74] and LMICs [41, 81, 83] that demonstrate similar convergent validity estimates with depression and anxiety. Previous studies have found large positive correlations between PTSD, depression, and anxiety [80, 83], such as a correlation of 0.78 between PTSD and depression [83]. This study also found strong relationships between PTSD and somatic symptoms, adding to a growing body of cross-cultural work suggesting the relevance of assessing somatic targets for traumatized populations [84, 85].

Certain items on the Hindi PCL-5 did not appear to measure PTSD in this cultural context. The hypervigilance item evinced nearly zero correlation with the remaining items on its arousal subscale, suggesting that it was not measuring PTSD-related arousal in this sample. Perhaps hypervigilance in the Indian slum context, where participants had to live more cautiously overall, did not function as an arousal symptom of PTSD in this setting. Slum communities in Bombay are characterized by frequent adversities, including home destruction by the government, lack of sanitation, lack of privacy for bathing and defecating, water and electricity shortages, and rat infestations [86]. It is possible that adaptive vigilance in a currently unsafe environment was confounded with hypervigilance symptoms linked to a prior traumatic event. Just over half the participants also reported ongoing stressors, including daily stressors, poverty-related strain, and current traumatic events. Experiencing ongoing stressors could elevate general stress and hypervigilance may not be linked with PTSD in this context. Alternatively, participants could have interpreted the item as appropriate—rather than maladaptive—vigilance, given previous study findings. The hypervigilance item, when translated literally into Portuguese for a study in Brazil [57], was also misunderstood by participants as a positive quality of appropriate alertness rather than the intended meaning of maladaptive hypervigilance. The hypervigilance item was also positively associated with alertness in another study in India (Charak et al. [78]). Taken together, these findings suggest that the hypervigilance item could have been interpreted by participants facing ongoing stressors as adaptive and therefore unrelated to other PTSD symptoms.

Trauma-related amnesia also evinced nearly zero correlation with the remaining items on the NACM subscale. Participants responded to this item by emphasizing that they could not *stop* thinking of their stressful experiences and ruminated on the details. This item also received the lowest endorsement, with only 14% of participants rating it at or above ‘2’ on a 0 to 4 scale. In other words, most participants (86%) reported no difficulty remembering important parts of the index trauma. Notably, trauma-related amnesia is endorsed less frequently in non-HIC cultural groups and it is considered an inconsistent item across cultures [25, 87, 88] and in India [89]. In contrast, “thinking a lot” or “thinking too much” has emerged as a prominent complaint in myriad cultural settings following trauma and adversity, supporting the notion that rumination is more cross-culturally common compared to trauma-related amnesia [90, 91]. The current study’s results extend the literature by confirming that trauma-related amnesia following trauma exposure are neither common nor indicative of the NACM symptom cluster of PTSD among Indian women from slums. Altogether, two items of the Hindi PCL-5 appear to operate distinctly in India and deleting both items increased the Hindi PCL-5’s reliability to 0.89. This finding suggests that the hypervigilance and trauma-related amnesia items do not measure PTSD in this cultural context.

### Reliability and validity of the idioms of distress scale

Idioms of distress are particularly relevant to assess given the problematic nature of two PTSD symptoms (i.e., trauma-related amnesia and hypervigilance) measured by the Hindi PCL-5. The idioms of distress scale yielded good reliability following deletion of one item about believing the traumatic event was deserved for being a woman. The highest mean rating assigned to idioms was for tension, believing the traumatic event was predestined, *sar mein garmi* (heat in the head)*, dil mein udasi* (sadness in the heart), and dizziness. Tension emerged as the most strongly endorsed idiom, which is consistent with the existing literature that tension is a common idiom across linguistic groups indicating depression and anxiety [21, 34, 92, 93]. Tension has been documented as a primary complaint by women reporting marital and interpersonal problems [33, 34, 93], which corresponds with the finding that participants endorsed interpersonal trauma largely by husbands and in-laws. The idiom of believing the traumatic event was predestined is consistent with findings from domestic violence survivors in South India [8]. Tichy et al. (2011) proposed that predestiny may be culturally ingrained as a function of India’s caste system legacy. Although legally abolished, the caste system still shapes social and occupational life for Indians, who tend to view their position in the socioeconomic strata as relatively stationary and unchangeable [94]. The intersectionality between one’s caste and gender can layer social disadvantages, such that women from ethnic and religious minority backgrounds (e.g., Dalits and Muslims in this study) may be likelier to endorse a traumatic event as predestined. This hypothesis is speculative and needs verification in future studies.

The somatic idioms, such as *sar mein garmi* (heat in the head)*, dil mein udasi* (sadness in the heart), and dizziness, are also consistent with the literature on somatization in India [35–37, 95]. These idioms’ popularity strengthens the notion that distress is experienced and expressed physically for this population and somatization of distress is commonly reported across many cultures following trauma exposure [25, 85, 96]. Altogether, the three most strongly endorsed idioms are distinct from the PTSD symptoms assessed using *DSM-5* criteria. The fact that these three idioms diverged from PTSD symptoms suggests their potential clinical utility as trauma-related idioms to assess, and target for treatment, for Indian women from slums. This finding, however, is tempered by the finding that endorsement patterns were at low levels of severity. The level of severity may be partly explained by sample characteristics, as this study recruited a community sample.

The idioms of distress scale also showed large positive correlations with depression, anxiety, and somatic complaints. Considering that these idioms indicate meaningful reactions related to depression, anxiety, and somatic complaints, it is possible that the idioms might also indicate clinically elevated distress. However, as the idioms of distress scale is a novel scale created for this study, future studies should validate it against clinician-administered interviews and measures of functional impairment to verify if the scale measures clinically elevated distress.

### Limitations and strengths

This study is limited by a small sample size and by use of referral sampling rather than random sampling. Women who were referred by others or self-referred to the study may systematically differ from other women exposed to GBV. Although a fair representation of women living in urban poverty in India [97], the study sample is not generalizable to Indians from other socioeconomic groups or geographical regions. Another limitation relates to translation. Efforts were made to counterbalance lexical translation with conceptual equivalence that retained the spirit of each PTSD item. Despite such efforts, using an expert committee consensus method in which multiple experts translate the same assessment and compare their translations [98] could enhance future validation procedures. Similarly, using more idiomatic translation or translation into the local vernacular language could improve local comprehension. Finally, this study is limited by a lack of sensitivity and specificity analyses because clinician-administered measures were not used in this study. Sensitivity and specificity analyses can improve diagnostic accuracy, so future studies should expand upon this work by validating the subscales of the adapted HTQ against clinician-administered interviews, including the idioms of distress scale.

Notwithstanding these limitations, this study also has strengths. This study is the first to investigate PTSD in an under studied population of Indian women from slums exposed to GBV. Published studies from Indian slums mirror the demographic characteristics of the current study sample [86], indicating our findings are applicable to women from urban Indian slums. This study also developed and tested a culturally sensitive assessment tool to screen broadly for all types of GBV in India. Correspondingly, this study is the first to validate a commonly used assessment tool for *DSM-5* PTSD symptoms in association with GBV for this population. This study is also one of the few to systematically record and report ongoing stressors in trauma-exposed populations from LMICs. Although more nuanced measures of ongoing stress are needed for future studies in India, the descriptive information about ongoing stressors better contextualized study results, such as why the hypervigilance item may have proved problematic. Finally, this study also improves upon psychometric studies set in LMICs by reporting multiple reliability and validity indicators for universal (i.e., PTSD) and culturally relevant reactions (i.e., idioms of distress). Notably, this study was conducted before the COVID-19 pandemic (i.e., 2018). Therefore, the effects of GBV and psychological distress during national lockdowns is not reflected in our study.

## Conclusions

The adapted HTQ is a reliable and culturally valid assessment that includes a comprehensive trauma screen for India, an assessment of ongoing stressors, universal trauma reactions (i.e., PTSD measured by the Hindi PCL-5), and culturally relevant trauma reactions (i.e., idioms of distress measured by a new scale). As certain PTSD items may not be relevant for Indian women from slums, assessing idioms of distress is doubly relevant. Findings from this assessment can inform which treatment targets to focus on and sections of this measure may be abbreviated for use depending on the assessment needs in question.

## Supplementary Information

**Additional file 1**. Adapted Harvard Trauma Questionnaire (HTQ) for Indian women from slums containing the PTSD Checklist-5 (PCL-5) - English version.

## Data Availability

The datasets used and/or analyzed during the current study are available from the corresponding author, Anushka Patel (apatel@hsph.harvard.edu), on reasonable request.

## Abbreviations

DSM-5: Diagnostic and Statistical Manual of Mental Disorders—5th edition
HIC: High-income country
HTQ: Harvard Trauma Questionnaire
GBV: Gender-based violence
LMICs: Low-and-middle-income countries
PCL-5: PTSD checklist for *DSM-5*
PTSD: Posttraumatic stress disorder
