# Supplementary material for: A pilot study adapting and validating the Harvard Trauma Questionnaire (HTQ) and PTSD checklist-5 (PCL-5) with Indian women from slums reporting gender-based violence
Source: BMC Womens Health. 2022 Jan 28;22:22. doi: 10.1186/s12905-022-01595-3 (PMC8795349; doi:10.1186/s12905-022-01595-3)
Supplement: Supplementary file 1 — Additional file 1. Adapted Harvard Trauma Questionnaire (HTQ) for Indian women from slums containing the PTSD Checklist-5 (PCL-5) - English version. [file 12905_2022_1595_MOESM1_ESM.pdf]

ADAPTED HARVARD TRAUMA QUESTIONNAIRE WITH PTSD CIVILIAN CHECKLIST -5

Indian Version

Name/ID: ____

Sex: ____

Date of Birth: ____

Marital Status: _______________________________________

Date: ____

Weathers, F.W., Litz, B.T., Keane, T.M., Palmieri, P.A., Marx, B.P., & Schnurr, P.P. (2013). *The PTSD Checklist for DSM-5 (PCL-5)*. Scale available from the National Center for PTSD at www.ptsd.va.gov.

Adapted from: Mollica, R. F., Caspi-Yavin, Y., Bollini, P., Truong, T., Tor, S., & Lavelle, J. (1992). The Harvard Trauma Questionnaire: Validating a cross-cultural instrument for measuring torture, trauma, and posttraumatic stress disorder in Indochinese refugees. *The Journal of nervous and mental disease*, *180*(2), 111-116.

Adaptation made by Anushka Patel (2015) at The University of Tulsa.

Part I

I am now going to ask you questions about stressful life events that sometimes happen to people. Some of these questions may feel sensitive but please answer yes or no if these events did or did not happen to you as honestly as you can. There are no right or wrong answers and everything you say will be kept confidential.

Event	Yes /No	Perpetrator	Pregnant	Age
**Interpersonal physical violence**				
Been hit, beaten or kicked?		Partner		
		Family member		
		Friend/Acquaintance		
		Stranger		
Been hit with an object/weapon/had something thrown at you (e.g. boiling water or acid)?		Partner		
		Family member		
		Friend/Acquaintance		
		Stranger		
Been threatened with violence?		Partner		
		Family member		
		Friend/Acquaintance		
		Stranger		
Witnessed someone being hit, beaten or kicked?		Partner		
		Family member		
		Friend/Acquaintance		
		Stranger		
Witnessed someone being hit with an object/weapon/something thrown at them (e.g. boiling water or acid)?		Partner		
		Family member		
		Friend/Acquaintance		
		Stranger		
Witnessed someone being threatened by violence?		Partner		
		Family member		
		Friend/Acquaintance		
		Stranger		
Personally experienced a robbery or mugging?		Partner		
		Family member		
		Friend/Acquaintance		
		Stranger		
Personally experienced police brutality when you did not commit a crime?		N/A		
Been involved in communal riots?		N/A		
**Sexual violence**				
Coerced into commercial sex work?		Partner		
		Family member		
		Friend/Acquaintance		
		Stranger		

Made to touch someone’s private parts when you did not want to?		Partner		
		Family member		
		Friend/Acquaintance		
		Stranger		
Had someone touch your private parts when you did not want them to?		Partner		
		Family member		
		Friend/Acquaintance		
		Stranger		
Made to have a hysterectomy or abortion?		Partner		
		Family member		
		Friend/Acquaintance		
		Stranger		
Had a physical or sexual assault that resulted in miscarriage or infant harm?		Partner		
		Family member		
		Friend/Acquaintance		
		Stranger		
Contracted a sexually transmitted disease from unwanted sex?		Partner		
		Family member		
		Friend/Acquaintance		
		Stranger		
Made to have sex with someone against your will?		Partner		
		Family member		
		Friend/Acquaintance		
		Stranger		
**Emotional violence**				
Been ignored by a loved one for a long period of time?		Partner		
		Family member		
		Friend/Acquaintance		
		Stranger		
Been called derogatory names for being a woman?		Partner		
		Family member		
		Friend/Acquaintance		
		Stranger		
Been called derogatory names for having a girl child?		Partner		
		Family member		
		Friend/Acquaintance		
		Stranger		
Been consistently humiliated or made to feel small by someone?		Partner		
		Family member		
		Friend/Acquaintance		
		Stranger		
Felt that someone else consistently made decisions for you?		Partner		
		Family member		
		Friend/Acquaintance		

		Stranger		
Learned about your husband having an affair?		N/A		
**Family-related violence**				
Experienced violence, coercion or bullying related to dowry?		Partner		
		Parent		
		In-laws		
		Other: _______________		
Were forced into an unwanted marriage?		Partner		
		Parent		
		In-laws		
		Other: _______________		
Personally experienced a family member hurting your child in order to hurt you?		Partner		
		Parent		
		In-laws		
		Other: _______________		
**Physical and mass trauma**				
Experienced starvation for an extended period of time?				
Survived a life-threatening medical illness?				
Survived a serious accident (e.g. house fire or car accident)?				
Survived a serious natural disaster (e.g. earthquake or flood)?				
**Bereavement or loss**				
Experienced forced separation from a child?				
Experienced forced separation from a family member?				
Experienced loss of a loved one due to unexpected or violent death?				
Experienced loss or destruction of home/land?				

Please specify any other situation that was very frightening or in which you felt your life was in danger:

Part II: Personal Description

Please indicate what you consider to be the most hurtful of terrifying events you have experienced. Please specify where and when these events occurred.

____

____

____

Under your current living situation, what is the worst event that has happened to you, if different from above. Please specify where and when these events occurred.

____

____

____

Part III: Trauma Symptoms

Below is a list of problems and complaints that people sometimes have after stressful life experiences. Please read each one carefully, and circle one of the numbers to the right to indicate how much you have been bothered by that problem in the past month.

0 = Not at all 1 = A little 2 = Moderately 3 = Quite a bit 4 = Extremely

	**PCL-5 symptoms**	**0**	**1**	**2**	**3**	**4**
1	Repeated, disturbing and unwanted memories of the stressful experience?					
2	Repeated disturbing dreams of the stressful experience?					
3	Suddenly feeling or acting as if the stressful experience were actually happening again (as if you were back there reliving it?)					
4	Feeling very upset when something reminded you of the stressful experience?					
5	Having strong physical reactions when something reminded you of the stressful experience (for ex. Heart pounding, trouble breathing, sweating)?					
6	Avoiding internal reminders of the stressful experience (ex. Thoughts, feelings or physical sensations)?					
7	Avoiding external reminders of the stressful experience (ex. People, places, conversations, objects, activities or situations)?					
8	Trouble remembering important parts of the stressful experience?					
9	Having strong negative beliefs about yourself, other people, or the world (ex. Having thoughts such as: I am bad, there is something seriously wrong with me, no one can be trusted, the world is completely dangerous)?					
10	Blaming yourself or someone else strongly for the stressful experience or what happened after it?					
11	Having strong negative feelings such as fear, horror, anger, guilt or shame?					
12	Loss of interest in activities that you used to enjoy?					
13	Feeling distant or cut off from people?					
14	Having trouble experiencing positive feelings (ex. Being unable to have loving feelings for those close to you, or feeling emotionally numb)?					
15	Feeling irritable or angry or acting aggressively?					
16	Taking too many risks or doing things that cause you harm?					
17	Being 'superalert' or watchful or on guard?					
18	Feeling jumpy or easily startled?					
19	Having difficulty concentrating?					
20	Trouble falling or staying asleep?					

The following are symptoms that people sometimes have after experiencing hurtful or terrifying events in their lives. Please read each one carefully and decide how much the symptoms bothered you in the past week.

1 = Not at all 2 – Q little 3 = Quite a bit 4 = Extremely

	**Culturally-salient symptoms**	**1**	2	3	4
1	Poor memory				
2	Feeling exhausted/fatigued				
3	Troubled by bodily pain or physical problems				
4	Feeling that you have less skills than before				
5	Thinking a lot				
6	Feeling unable to make daily plans				
7	Having difficulty dealing with new situations				
8	Feeling that you are the only one who suffered these events				

9	Feeling that others don't understand what happened to you				
10	Feeling guilty for having survived				
11	Blaming yourself for things that have happened				
12	Spending time thinking why God is making you go through such events				
13	Feeling a need for revenge				
14	Feeling others are hostile towards you				
15	Feeling that someone you trusted betrayed you				
16	Feeling no trust in others				
17	Feeling that you have no one to rely on but God				
18	Hopelessness				
19	Feeling powerless to help others				
20	Feeling ashamed of the hurtful or traumatic events that have happened to you				
21	Feeling humiliated by your experience				
22	Feeling that you are a jinx to yourself and your family				
23	Finding out or being told by others that you have done something that you can't remember				
24	Feeling as if you are watching yourself and your actions from the outside				
	**Cultural idioms of distress**				
1	Naara mein dard (pain in the nerves)				
2	Sar mein garmi (heat in the head)				
3	Chakkar (dizziness)				
4	Dhatu/Safedpaani (leukorrhea)				
5	Tension				
6	Dil mein udasi (sadness in your heart)				
7	Fear of others learning about this hurtful experience				
8	Believing that this was destined to happen and written in your stars				
9	Feeling that you are a burden on your family				
10	Feeling that you brought shame on your family				
11	Believing that you deserved what happened because you are a woman				
12	Thinking a lot				
13	Dizziness when standing up				
